# Lactobacillus oris improves non-alcoholic fatty liver in mice and inhibits endogenous cholesterol biosynthesis

**DOI:** 10.1038/s41598-023-38530-x

**Published:** 2023-08-09

**Authors:** Yiming Zhao, Congyong Li, Zhe Luan, Jun Chen, Cong Wang, Yujia Jing, Shirui Qi, Zhizhuang Zhao, Hanwen Zhang, Junling Wu, Yi Chen, Zhuanyu Li, Bowen Zhao, Shufang Wang, Yunsheng Yang, Gang Sun

**Affiliations:** 1 Department of Gastroenterology and Hepatology, Hainan Hospital of PLA General Hospital, Sanya, 572013 China; 2 Sixth Health Care Department, Second Medical Center of PLA General Hospital, Beijing, 100853 China; 3 Department of Gastroenterology and Hepatology, First Medical Center of PLA General Hospital, Beijing, 100853 China; 4 Unit 91917, Beijing, 102401 China; 5 Emergency Department, Tianjin First Central Hospital, Tianjin, 300192 China; 6 Beijing QuantiHealth Technology Co., Ltd., Beijing, 100070 China; 7 Institute of Medicinal Plant Development, Chinese Academy of Medical Sciences and Peking Union Medical College, Beijing, 100193 China

**Keywords:** Microbiology, Gastroenterology, Medical research

## Abstract

We previously confirmed that a strain of Lactobacillus oris isolated from the fecal samples of healthy Hainan centenarian having potent lipid-lowering ability in HepG2 cells; and this study was to investigate the effect of the stain on non-alcoholic fatty liver in mice in vivio. The Lactobacillus oris strain isolated from Hainan centenarian fecal samples were frozen stored in our laboratory. Thirty ob/ob mice (10 in each group) were orally gavaged with Lactobacillus oris (Lactobacillus, 5 × 10^9^ cfu), mixed probiotics (Mixed, 5 × 10^9^ cfu, a mixture with known lipid-lowering ability), or culture medium (Control) respectively. Lactobacillus oris isolated from fecal samples of Hainan centenarians showed significantly in vivo lipid lowering ability compared with the controls, and the ability was comparable with mixed probiotics strains in mice The possible mechanisms of lipid-lowering of probiotics and Lactobacillus oris may be associated with HMGR inhibition to suppress the synthesis of endogenous cholesterol; bile acids reabsorption, and intestinal FXR-FGF15 signaling pathways promoting the cholesterol conversion into bile acids secretion.

## Introduction

Nonalcoholic fatty liver disease (NAFLD) refers to the liver conditions of extra fat buildup in people who drink little or no alcohol (the nomenclature of NAFLD has been suggested to change to Metabolic Associated Fatty Liver Disease “MAFLD” to more accurately reflect pathogenesis by an international panel of experts^1^. NAFLD usually causes no signs or symptoms but is associated with a significantly higher overall mortality rate compared to the general population^2,3^. NAFLD is becoming increasingly common worldwide, with a global prevalence of approximately 25–30%, and about 3.6 million new cases each year^4,5^. The etiology of NAFLD involves both external and internal factors, including genetic susceptibility, lifestyle, environment, diet, and gut microbiota. Some lifestyle changes can control or reverse the fat buildup in the liver, but not all patients respond in the same manner^6,7^; Mediterranean diet and physical exercise are recommended as a treatment of NAFLD targeting at weight loss of 5–7%^8^; and liraglutide, pioglitazone or vitamin E are effective in the treatment of nonalcoholic steatohepatitis, but with serious adverse events^9^. Currently, there are no approved safe and effective treatments for NAFLD. Therefore, new treatments for NAFLD are emergently needed in clinical practice.

The gut microbiota (GM) is the microbial community existing in the human gastrointestinal tract, which is considered a virtual organ of the human body. GM hosts approximately 100 trillion microorganisms, and the microbiome has 100 times as many genes as the human genome. GM affects host fitness, phenotype, and health, and plays a role in the development and progression of many diseases, including NAFLD^7,10^. The bile acid metabolism and hepatic fat deposition are regulated by gut microbial enzymes and gut microbiota dysbiosis^11^. Probiotics are generally considered safe food supplements and have shown promising effect in restoring gut flora and improving NAFLD. A single probiotic strain can be used as a monotherapy, or a few probiotics can be used in combination^12–14^. Compared with other lipid-lowering drugs, probiotics have the advantages of high safety, few side effects, and short production cycle.

Hainan, a southern province of China and the most populous island, is known for the long-life expectancy and high number of centenarians. Longevity is a result of a variety of factors, such as unique eating habits, diet, and different basal metabolic rate, which may also lead to changes in GM. GM of centenarians may have the potential to counteract fat deposition and improve fat metabolism. Therefore, we isolated and identified probiotic strains with lipid-lowering potential from the fecal samples of the centenarians. Previously, we established a gut microbial bank containing more than 8000 strains of gut bacteria and more than 1200 potential probiotics based on samples from 1473 centenarians in Hainan (accounting for more than 80% of the island's centenarians)^15^. Ten probiotics with unique evolutionary directions (presented by different pattern of branching in the phylogenetic tree) were obtained through in-depth metagenomic sequencing, single nucleotide polymorphisms and culturomics methods. Lactobacillus oris was identified to have in vitro lipid-lowering ability in HepG2 cells in our previous unpublished work. In this study, we tended to test the in vivo lipid-lowering efficacy of Lactobacillus oris using ob/ob mice^16^.

## Results

### Characteristics of lactobacillus oris strains

The purified strains were identified based on their morphological features and biochemical properties. This strain was positive for Gram staining and could grow at temperatures as low as 20 °C. Catalase test, motility test, nitrate reduction, H2S production, and gelatin liquefaction tests were all negative. It was preliminarily identified as Lactobacillus oris. The whole-genome sequencing confirmed that it was Lactobacillus oris, and the sequence data has been deposited in NCBI GenBank with accession number OP132831. The mixed probiotics were isolated from the same fecal samples. These strains were with known lipid-lowering potentials. Their sequence information has been deposited in GenBank : *Lactobacillus gasseri* (accession no: OP132582)*, Lactobacillus fermentum* (OP132583)*, Lactobacillus paracasei* (OP132829)*, Bifidobacterium breve* (OP132527)*, Bifidobacterium longum* (OP132828).

### Weight gain and liver weight

**Figure 1:**
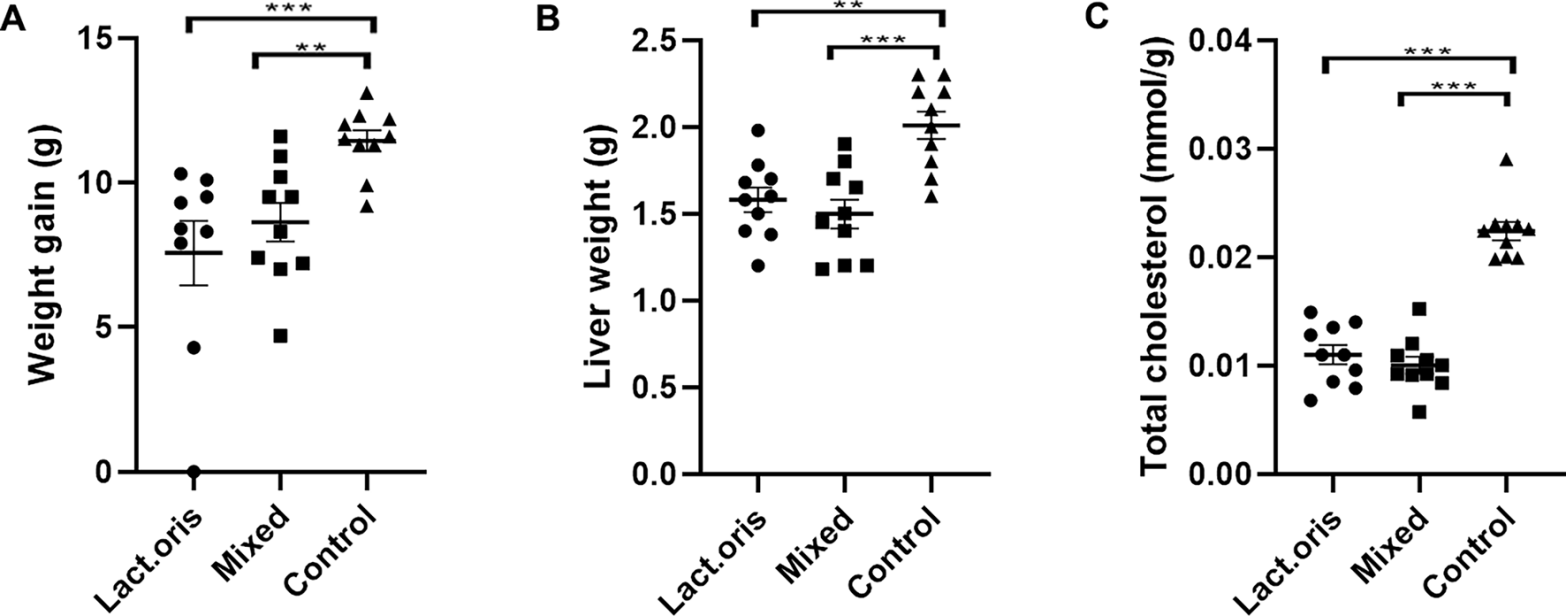
(**A**) weight gain of the treated mice and control mice, and (**B**) liver weight of the treated mice and control mice at the end of the fifth week; (**C**) total cholesterol levels of treated mice and control mice; ** indicates *P* < 0.01, *** indicates *P* < 0.001.

The mouse body weight gain is shown in Fig. 1A, the liver weight is shown in Fig. 1B, and the total cholesterol content is shown in Fig. 1C. At the end of the fifth week of treatment, the weight gain of the mice in both the Lactobacillus oris treatment group and the mixed probiotics treatment group were significantly reduced (*P* < 0.01, *P* < 0.001). At the end of the fifth week of treatment, the liver weight and the total cholesterol were significantly reduced in both treatment groups (Lactobacillus oris group and mixed probiotics group) compared with the control group (*P* < 0.01, *P* < 0.001; *P* < 0.001, *P* < 0.001).

### H&E staining and oil red O staining of liver tissues

**Figure 2:**
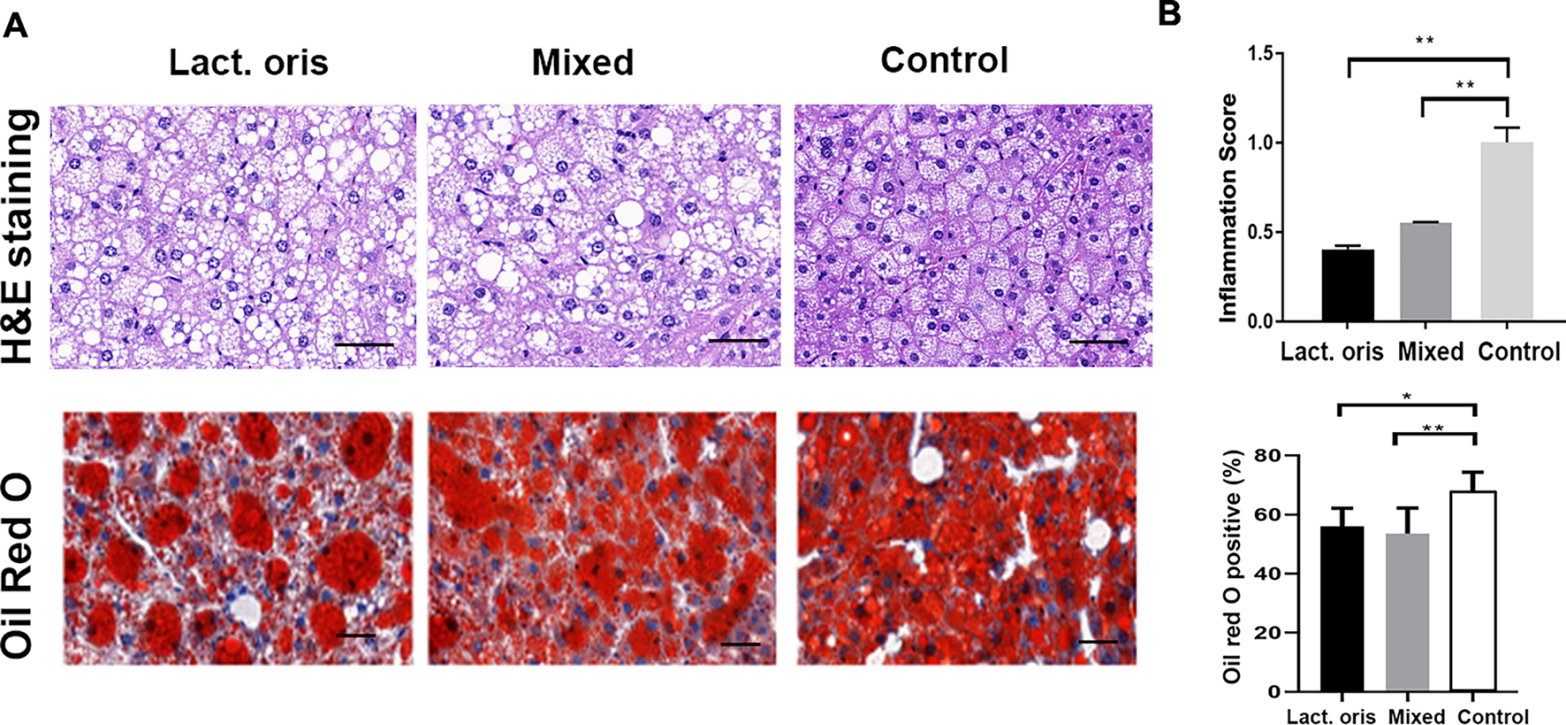
(**A**) H&E staining and oil red O staining of mouse liver tissue, and (**B**) Liver inflammation score and percentage of fat vacuoles in liver tissue; scale bar = 25 µm * indicates *P* < 0.05, ** indicates *P* < 0.01.

The results of H&E staining and oil red O staining of mouse liver tissues are shown in Fig. 2. Microsteatosis was observed in H&E-stained liver biopsies from both treated and control mice. There were visible fatty vacuoles, loss of liver lobular structure, and inflammatory cell infiltration. Compared with the control group, the liver steatosis and inflammatory cell infiltration of the mice in the Lactobacillus oris treatment group and the mixed probiotics treatment group were significantly reduced (*P* < 0.01). Oil Red O staining results showed that compared with the control group, the percentage of fat vacuoles in the Lactobacillus oris treatment group and the mixed probiotics treatment group was significantly reduced (*P* < 0.05, *P* < 0.01).

### AST and ALT levels

**Figure 3:**
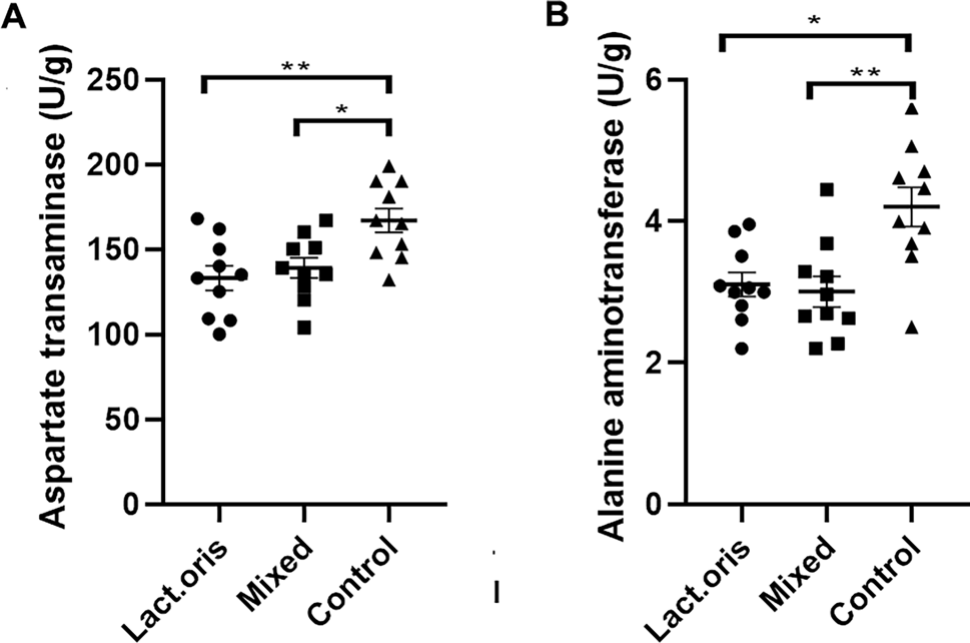
(**A**) aspartate transaminase level, (**B**) alanine aminotransferase level, and * indicates *P* < 0.05, ** indicates *P* < 0.01.

As shown in Fig. 3, after consecutive oral gavage for five weeks, the liver AST and ALT levels of the Lactobacillus oris treatment group and the mixed probiotics treatment group were significantly lower than those of the control group.

### RNA expression and real-time RT-PCR

**Figure 4:**
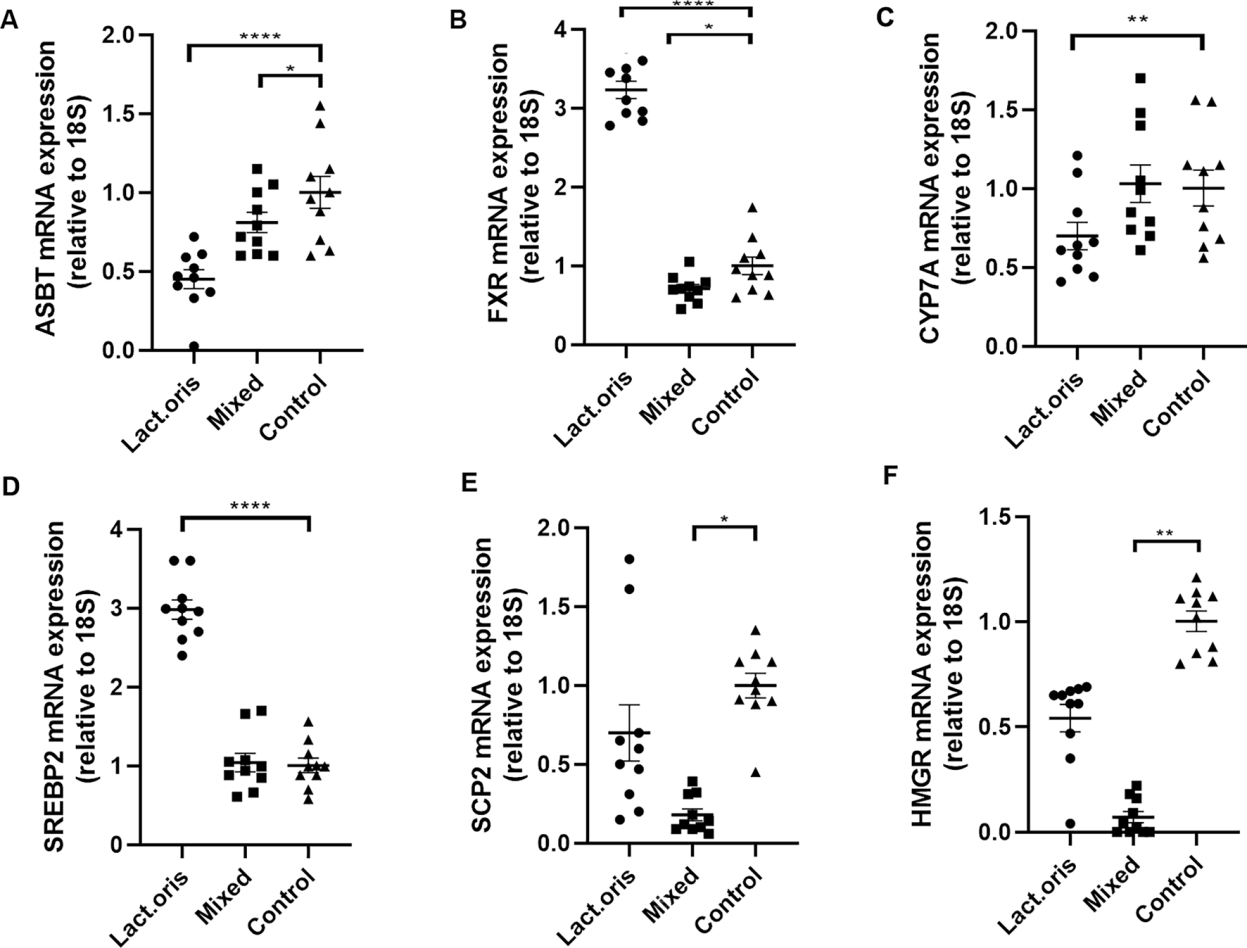
The RNA expressions: **(A)** apical sodium-dependent bile acid transporter in the intestine, (**B**) Farnesoid X receptor in the intestine, (**C**) CYP7A in the liver, (**D**) SREBP2 in the liver, (**E**) SCP2 in the liver, and (**F**) HMGR in the liver of treated and control mice. * indicates *P* < 0.05, ** indicates *P* < 0.01, *** indicates *P* < 0.001.

The RNA expressions are shown in Fig. 4A and B: intestine; C–F: liver. Compared with the control mice, the Lactobacillus oris treated mice had significant higher levels of FXR and SREBP2 mRNA and significant lower levels of ASBT and CYP7A mRNA (*P* < 0.05). Compared with the control group, the mixed probiotics treated mice, but not the Lactobacillus oris treated mice, had lower expressions of HMGR and SCP2 mRNA (*P* < 0.05).

## Discussion

Currently, there are no medicines approved for the treatment of NAFLD. NAFLD management includes weight loss, exercise and dietary supplementation, and lipid-lowering or cholesterol-lowering medicines are used clinically to treat NAFLD. Probiotics are believed can improve NAFLD, but the exact underlying mechanism is unclear^17,18^. In this study, we have tested the in vivo lipid-lowering ability of lactobacillus oris through gavage the strain into ob/ob mice, and explored the possible mechanism based on the expression of endogenous cholesterol biosynthesis pathways. The source of the lactobacillus oris was fecal samples of centenarians in Hainan province. Centenarians are a special population who live healthy into their 100 s and may have overcome metabolic syndrome disorders such as NAFLD. We have previously isolated and identified lactobacillus oris from fecal samples of centenarians in Hainan as strains with in vitro lipid-lowering ability in HepG2 cells experiments. We compared the effect of lactobacillus oris with a mix of probiotics as the positive control, a product of VSL Pharmaceuticals, Ft. Lauderdale, FL), that had been extensively tested in animal models of intestinal inflammation^8^.

The gut microbiota supports the growth of specialized microbes that produce short chain fatty acids (SCFAs) including acetate, propionate, and butyrate^10^. Butyrate is the main energy source for human colonocytes; propionate is transferred to the liver to regulate gluconeogenesis and satiety signaling; acetate, the most abundant SCFA, is used in cholesterol metabolism and lipogenesis, and may play a role in central appetite regulation^4^. However, the interaction between gut microbiota regulation and bile acid regulation on liver lipid deposition remains elusive. The bile salt hydrolase of probiotics is thought to play an important role in bile acid homeostasis because it hydrolyzes the bound bile salts to form amino acids and less soluble free bile acids, which bind to cholesterol to reduce serum cholesterol levels^19,20^. Bile acids metabolism play a vital role in hepatic fat deposition. The enterohepatic circulation of bile acids from the liver to the intestine and back to the liver regulates nutrient absorption and homeostasis^5,21^. Bile acid feedback inhibition of its own synthesis has been studied for decades, but the underlying molecular mechanism remains unclear. Bile acids may participate in the regulation of gene transcription because they are endogenous ligands for the nuclear receptor Farnesoid X receptor (FXR). FXR agonists have been used to treat nonalcoholic fatty liver disease (NAFLD), in part because they reduce hepatic lipids^22^. Sterol regulatory element-binding proteins (SREBPs, including SREBP1a, SREBP1c, and SREBP2) are basic-helix-loop-helix leucine zipper (bHLH-Zip) transcription factors that regulate the synthesis and cellular uptake of cholesterol and fatty acids^23^. In our study, Lactobacillus oris treatment increased the expressions of both FXR and SREBP-2 in the liver of mice, suggesting that it might improve NAFLD through the FXR and SREBP pathways. Changes in the sodium-dependent bile acid transporter (ASBT) in the apical ileum have been shown to affect the size of the bile acid pool^24^; and rate-limiting enzyme cholesterol 7α-hydroxylase (CYP7A) and 3-hydroxy-3-methylglutaryl CoA reductase (HMGR) mRNAs are also involved in rat liver cholesterol modulation through bile acid pathways^25^. Consistent with other studies, both intestine ASBT and liver CYP7A were reduced by Lactobacillus oris, while this CYP7A lowering effect was not observed in the mixed probiotics treatment, indicating that the lipid-lowering mechanism of Lactobacillus oris and the mixed probiotics may be slightly different.

The study has limitations. First, the ob/ob mice have a mutation in the leptin gene and increase appetite, and progressive food intake leads to the development of severe obesity and fatty livers. Therefore, genetically defective OB/OB mice may not fully model fat metabolism in normal mice. On the other hand, weight gain in the body and fat accumulation in the liver may be more easily observed than in normal mice. Second, not using lean + / + littermate controls may compromise the statistical conclusion validity. Third, the study did not provide the markers of insulin resistance and glucose tolerance, which would add significance to the results. Future studies should select more appropriate models and provide more complete data to better understand the potential of probiotics to improve NAFLD.

## Conclusions

The Lactobacillus oris strain isolated from Hainan centenarian fecal samples showed significantly in vivo lipid lowering ability compared with the controls, and the ability was comparable with mixed probiotics strains. The possible mechanisms of lipid-lowering of probiotics and Lactobacillus oris are associated with inhibition of HMGR to suppress the synthesis of endogenous cholesterol; bile acids reabsorption, and intestinal FXR-FGF15 signaling pathways promoting the cholesterol conversion into bile acids secretion.

## Methods

### Animals

Thirty male C57BL-6 ob/ob mice (4 weeks old, weighing 34.53 g) were obtained from Huafukang Bio, Beijing (License No: SCXK(Jing) 2019-0008). They were group-housed (5 animals per cage) in a control environment with a 12:12-h light–dark cycle^26,27^. The ob/ob mice have a mutation in the leptin gene and increased appetites, and they tend to develop severe obesity and fatty livers. Mice were provided with water and a standard chow diet (nutritional characteristics of the diet by weight: water ≤ 10%, crude protein ≥ 18%, crude fat ≥ 4%, crude fiber ≤ 5%, cholesterol = 0%) ad libitum throughout the experiment. The study protocol was approved by the Laboratory Animal Ethics Committee of the Chinese People’s Liberation Army General Hospital (No.: SQ2020023).

### Source and identification of bactria stains

Lactobacillus oris was isolated from the fecal samples of 75 healthy Hainan centenarians and stored in our laboratory. Briefly, the sample donors were healthy, had a normal diet, and were not treated with antibiotics. The written consent was obtained from each sample donor. The purification, culturing, and identification by MALDI-TOF mass spectrometry were performed as previously described^28^. The whole genome sequencing of bacterial DNA was performed by Beijing Quantitative Health Technology Co., Ltd as described previously^29^. The mixed probiotics with known lipid-lower ability were isolated from the same fecal samples as Lactobacillus oris and stored in our laboratory till the use.

### Preparation of gavage solution

The strains were frozen at − 80 °C, streaked on the solid MRS medium by the zigzag streak method, and cultured in an anaerobic workstation (10% H_2_ + 10% CO_2_ + 80% N_2_) at 37 °C for 48 h to obtain single colonies. A single colony was inoculated into 5 mL of liquid MRS medium in an anaerobic workstation (10% H2 + 10% CO_2_ + 80% N_2_) for 24 h at 37 °C to obtain seed liquid. Inoculate the seed solution (1% inoculum amount) to the MRS liquid culture medium anaerobic workstation (10% H_2_ + 10% CO_2_ + 80% N_2_) for 24 h at 37 °C to obtain the working bacterial solution. The working bacterial solution was subjected to a ten-fold gradient dilution, and the viable bacterial count was recorded. Based on the bacterial concentration required for gavage, the working bacterial solution was diluted and concentrated to obtain a gavage culture solution, which was transferred to an anaerobic tube and stored under refrigeration for future use.

### Treatment regimen

Thirty male C57BL-6 ob/ob mice were divided into three groups, 10 in each group. Each group received daily oral gavage of Lactobacillus oris strains (5.0 × 10^9^ CFU/ml, 0.03 ml/g), the mixed probiotics with approved lipid-lowering effect (5.0 × 10^9^ CFU/ml, 0.03 ml/g, the mixed strains include *Lactobacillus gasseri, Lactobacillus fermentum, Lactobacillus paracasei, Bifidobacterium breve, Bifidobacterium longum*), or culture medium (YCFA, 0.03 ml/g) respectively for 5 weeks. At the end of treatment, mice were anesthetized by intraperitoneal injection of sodium pentobarbital, the retro-orbital bleeding was performed to collect the blood, and then mice were killed by neck dislocation. Liver tissue and small intestine tissue were obtained.

### Biochemistry & histology of the liver

During treatment, the activities of alanine aminotransferase (ALT), aspartate transaminase (AST), and total cholesterol (TC) in serum were measured. The TC assay kit was purchased from Nanjing Jiancheng bioengineering institute.

Mouse liver tissue specimens were fixed in 4% paraformaldehyde solution for 24 h and embedded in paraffin. The specimens were cut into 5 mm sections for H&E staining and oil red O staining. After oil red O staining, the grade of liver steatosis was observed under a microscope. The liver inflammation scores were assessed by METAVIR method, and the activity grade indicates the activity or degree of inflammation, namely, A0: no activity; A1: mild activity; A2: moderate activity; and A3: severe activity. The average score was compared between groups.

### Real time-PCR assessment for endogenous cholesterol synthesis

RNA was extracted from mouse liver tissue and small intestine tissues by RNAiso Plus (Trizol) and reverse transcribed into cDNA. Primers were designed and specific primers were determined by BLAST analysis. β-actin was used as the internal reference. The primer sequences used are: FXR forward: GTGACAAAGAAGCCGCGAAT; reverse: GCAGGTGAGCGCGTTGTAAT; FGFR4 forward: GGTCCTCTGGCAAGTCAAGT; reverse: CTCCCAAAGTGGATCGAGTTGG; SRAGEB2 forward: TCAGACATTCGAGTTGGATGGATGCGTAG; CATACCCAATGTGCCTGGAT; Reverse: AACCAAGCCACCCTACCTG; CYP7A1 forward: -GCTATTCTCTGGGCATCTCAAG reverse: GAAAGTCAAAGGGTCTGGGT. The cycle protocols are: pre-denaturation at 95 °C for 60 s; denaturation at 95 °C for 15 s, annealing at 60 °C for 15 s, and extension at 72 °C for 45 s; 40 cycles. The reverse transcription reaction kits and real-time PCR kits were purchased from Takara biomedical technology (Beijing) Co., Ltd; primers were synthesized by Shangon Biotech (Shanghai) Co., Ltd. The relative expression of the target-gene was calculated by qPCR using the delta-delta Ct (2^−∆∆Ct^) method.

### Statistical analysis

The experimental data are expressed as mean ± standard deviation. A one-way analysis of variance (ANOVA) or t-test was used for statistical analysis of the data. *P* < 0.05 was considered to be statistically different. Graphpad prism version 8 statistical software was used for analysis and graph generation (Supplementary Information 1).

### Ethical approval

The study protocol was approved by the Experimental Animal Ethics Committee of the Chinese People’s Liberation Army General Hospital (No.: SQ2020023) The study was carried out in compliance with the ARRIVE guidelines. All methods were carried out in accordance with relevant guidelines and regulations.

### Supplementary Information

Supplementary Table 1.

## Data Availability

The sequence data has been deposited at the NCBI Sequence Read Archive (SRA) database (BIOProject: PRJNA772518). The datasets used and/or analyzed during the current study are available from the corresponding author on reasonable request.

## Abbreviations

ASBT: Apical sodium-dependent bile acid transporter
NAFLD: Nonalcoholic fatty liver disease
MAFLD: Metabolic associated fatty liver disease
GM: Gut microbiota
TC: Total cholesterol
ALT: Alanine aminotransferase
AST: Aspartate transaminase
