# Supplementary material for: Lactobacillus oris improves non-alcoholic fatty liver in mice and inhibits endogenous cholesterol biosynthesis
Source: Sci Rep. 2023 Aug 9;13:12946. doi: 10.1038/s41598-023-38530-x (PMC10412569; doi:10.1038/s41598-023-38530-x)
Supplement: Supplementary file 1 — Supplementary Table 1. [file 41598_2023_38530_MOESM1_ESM.docx]

S1: individual mouse weight of the Lactobacillus oris group

| Mouse code | 20190801 weight/g | 20190808 weight/g | 20190815 weight/g | 20190822 weight/g | 20190829 weight/g | 20190905 weight/g |
| --- | --- | --- | --- | --- | --- | --- |
| A1-1 | 35.10 | 33.70 | - | - | - | - |
| A1-2 | 35.30 | 34.30 | - | 36.50 | 38.00 | 39.60 |
| A1-3 | 32.80 | 35.30 | 36.70 | 37.00 | 39.40 | 41.20 |
| A1-4 | 34.80 | 37.80 | 38.00 | 39.30 | 41.50 | 44.10 |
| A1-5 | 36.50 | 38.40 | 40.90 | 43.00 | 45.00 | 44.40 |
| A2-1 | 34.30 | 37.10 | 39.30 | 41.70 | 43.40 | 44.60 |
| A2-2 | 35.70 | 38.40 | 38.10 | 41.20 | 43.90 | 45.80 |
| A2-3 | 34.70 | 36.50 | 38.80 | 40.80 | 42.90 | 44.20 |
| A2-4 | 36.50 | 39.20 | 41.00 | 42.40 | 43.60 | 44.80 |
| Mean | 35.08 | 36.74 | 38.97 | 40.24 | 42.21 | 43.59 |

S2: individual mouse weight of the mixed probiotics group

| Mouse code | 20190801 weight/g | 20190808 weight/g | 20190815 weight/g | 20190822 weight/g | 20190829 weight/g | 20190905 weight/g |
| --- | --- | --- | --- | --- | --- | --- |
| B1-1 | 35.00 | 37.10 | 36.40 | 38.00 | 40.00 | 42.00 |
| B1-2 | 34.30 | 36.90 | 39.10 | 40.60 | 42.80 | 43.80 |
| B1-3 | 34.90 | 38.40 | 40.30 | 42.40 | 41.70 | 42.10 |
| B1-4 | 34.20 | 36.00 | 37.90 | 38.90 | 40.20 | 41.60 |
| B1-5 | 34.00 | 34.50 | 36.60 | 38.70 | 40.60 | 42.30 |
| B2-1 | 33.70 | 35.70 | 38.40 | 41.50 | 43.60 | 45.30 |
| B2-2 | 35.60 | 38.30 | 40.60 | 42.30 | 44.20 | 45.80 |
| B2-3 | 35.00 | 36.20 | 38.30 | 41.10 | 42.80 | 44.50 |
| B2-4 | 34.50 | 36.40 | 34.80 | 35.40 | 35.40 | 39.20 |
| B2-5 | 34.20 | 37.70 | 39.70 | 41.40 | 43.10 | 45.10 |
| Mean | 34.54 | 36.72 | 38.21 | 40.03 | 41.44 | 43.17 |

S3: individual mouse weight of the control group

| Mouse code | 20190801 weight/g | 20190808 weight/g | 20190815 weight/g | 20190822 weight/g | 20190829 weight/g | 20190905 weight/g |
| --- | --- | --- | --- | --- | --- | --- |
| D1-1 | 34.10 | 38.20 | 36.40 | 40.60 | 43.00 | 46.40 |
| D1-2 | 34.50 | 38.40 | 40.80 | 43.00 | 44.50 | 45.80 |
| D1-3 | 34.60 | 38.30 | 41.00 | 42.90 | 44.90 | 46.80 |
| D1-4 | 34.20 | 37.60 | 40.30 | 42.30 | 44.30 | 45.50 |
| D1-5 | 35.60 | 39.30 | 41.90 | 44.60 | 45.90 | 48.70 |
| D2-1 | 34.60 | 37.30 | 39.70 | 42.20 | 44.00 | 46.10 |
| D2-2 | 34.20 | 37.90 | 40.50 | 42.20 | 44.00 | 46.20 |
| D2-3 | 36.90 | 37.00 | 37.90 | 42.20 | 44.50 | 46.10 |
| D2-4 | 36.20 | 39.50 | 41.90 | 44.00 | 45.50 | 47.80 |
| D2-5 | 34.30 | 34.00 | 35.80 | 39.40 | 41.60 | 44.20 |
| Mean | 34.92 | 37.75 | 39.62 | 42.34 | 44.22 | 46.36 |
